# Twenty-Sixth Annual Session of the Alabama Dental Association, 1895

**Published:** 1895-08

**Authors:** J. M. Walker


### PROCEEDINGS.

### Twenty-sixth Annual Session of the Alabama Dental Association, 1895.

Reported for the DENTAL REGISTER by Mrs. J. M. Walker.

[*Continued from page* 306.]

The Association was called to order on the third day at 10 A. M.

Under the heading "Theory and Practice," the Secretary read a paper from Dr. C. L. Boyd, entitled "Something of What Dentists Do, and What They Should Do." The paper treated of the causes of failure in various branches of dental treatment, and outlined the remedies therefor, dwelling particularly upon the importance of the care of the teeth of children. The paper was discussed by Drs. Brown, Young, Crossland, Allen, Foster, Sann, Pearson and Prof. Gray.

The almost universal early failure of proximal fillings in molars and bicuspids, Dr. Boyd attributes to one or all of three causes: first, that the dentist is incompetent to handle the material he uses; second, that he fails to properly prepare the cavity, or third, that the teeth are of poor structure.

The causes of the numerous broken plates we are called on to repair was also discussed, the general causes being lack of foresight in adaptation and construction of the piece and in mal-occlusion.

Dr. Boyd dwelt feelingly upon the negligence of which children are the victims, through the ignorance of parents as to the value of their teeth, and how to care for them, insisting upon early dental attention and proper nourishing food. The greatest work of the dentist is to educate the people, teach them to be cleanly in the mouth, and to care for the children's teeth. He also strongly advises gum-massage, aiding the development of the jaw and preventing that crowding of the teeth which conduces to decay.

In the discussion of the paper Dr. Brown cited some cases of cement fillings in molars and bicuspids exposed to mastication, which were as hard as porcelain almost, having been in service from seven to twelve years. The durability of cement, he thinks, depends not so much on the make as in the method of manipulation, and also on the secretions of the mouth. It requires a certain knack which cannot be described, and also upon not overworking it—not working it after it begins to crystallize.

Dr. R. C. Lowry, judging by his own experience, considers cement as poor a thing as can be put in the mouth. He related two cases of aluminum crowns set with cement, the cusps being filled with amalgam, which melted down in the mouth in twelve hours.

Dr. Allen thinks that the trouble with cement, as with amalgam, lies in the fact that being so easy to insert, no care is taken in the preparation of the cavity. The cavity should be as carefully prepared and with all the same antiseptic and other precautions as though for gold, the filling material as carefully inserted, and the filling as thoroughly finished. Then you will get good results, no matter what you use. All deep cavities should be partially filled with cement—a good sticky cement—packing gold right into it. Then when this has hardened, trim the gold and condense it; this is better than retaining points. Dr. Allen does not approve of giving little children coarse, hard food to masticate. The temporary teeth are not suited to that purpose from their brief duration, during the early part of which the roots are undeveloped, and the teeth not firmly fixed in the alveolus, while during the latter portion of their retention the roots are being absorbed, the edges having sharp, jagged points not fitted to bear the pressure of excessive mastication. The effort being painful, the child swallows its food as best it can, and the foundation for dyspepsia is early laid. The first molars have the same imperfectly developed roots and the structure is immature and poorly calcified for some time after eruption. For this reason, if decay sets in early in these teeth, as is often the case, do not fill with gold, no matter how able and willing the parents may be to pay for gold, and do not use amalgam which discolors the dentine.

Use the cements or gutta-percha and renew as frequently as necessary, explaining to the parents the reasons for this course. In this way you will save the teeth, at least, until the function of the pulp is fulfilled. Use every effort to prevent early devitalization of the sixth-year molars while the root is incomplete. Application of the usual agents will be followed by trouble, because of the open funnel-shaped apex. A knowledge of physiology, histology and embryology is necessary to the dental operator if he would avoid serious blunders. Even for the making of a rubber plate a knowledge of physiology is necessary, as not every mouth can wear a red rubber plate, but I beg you will not discuss that point.

Prof. Gray has recently adopted a very satisfactory method of using gutta-percha. After drying the cavity he saturates it with common resin cut in chloroform and then presses in heated gutta-percha. It adheres to the walls like cement and does not pull away. He has found it very satisfactory in the mouths of his own children where he has the opportunity of observing it closely.

Dr. S. W. Foster: Dr. Boyd places before us two essential points—the prevention of caries, and the arrest of caries. One of the great causes of caries is the lack of hygienic care. With but few exceptions where the teeth are really kept clean there is but little caries. Another important consideration with young children is the preservation of equilibrium in development, physical and mental, with no overdevelopment in either direction. Do not confine them too closely in schools rooms on the one hand, and do not let them run with the pigs on the other.

Many of the failures in the proximal fillings mentioned by Dr. Boyd are due to carelessness in the preparation of the cavity margins. It is important to cut the walls well back and knuckle the fillings, otherwise the teeth will disintegrate midway between the gingival margin and the coronal surface. We also fail to make sufficient space for restoration of contour and knuckling, letting only metal come in contact—these cavities usually coming in pairs. If necessary, take a week to secure space (I use cotton for separating). It is the only way to succeed with these cavities.

Dr. Vann spoke of the method taught by Dr. Land, of

Detroit, and endorsed by many dentists of Chicago, of saturating the cavity walls with the liquid of the cement preparation, and then burnishing in the cement powder, as also the alloys. It made fillings that were like ivory, *out of the month*, but he had met with many failures in practice, after adopting the method which he learned in Chicago.

DR. PEARSON thinks the reason we see so many failures in amalgam fillings is because we put in such a large proportion of them. In his own practice he is compelled to put in ten amalgam to one of gold. His patients won't pay ten dollars for a gold filling when they can get amalgam for two.

DR. ALLEN: You ought to get six to eight dollars for an amalgam filling such as you would get ten for if gold!

Under the same heading, "Theory and Practice," DR. R. C. YOUNG read a paper, covering a large range of practice in operative dentistry. In obscure cases where neuralgia of the fifth pair of nerve leads to the suspicion of a dead tooth, which is difficult of location, Dr. Young suggests applying a ball of heated red gutta-percha to the teeth one after the other. When the right one is reached the response will be unmistakable, the localized heat causing an increased flow of blood to the already congested pulp. In case of great soreness of a tooth from periamentitis, he suggests a strong ligature tied around the tooth with long ends upon which the patient is directed to pull strongly away from the socket, up or down as the tooth may be an upper or lower tooth. This affords great relief while the tooth is being drilled into for the escape of the accumulated gasses. Disarticulate the tooth, and give it rest, by molding gutta-percha over an adjacent tooth, dropping it into a glass of cold water to restore its elasticity before placing permanently in position. After the pulp has been removed Dr. Young washes out with pyrozone 3 per cent, dry out and dress with beachwood creosote on cotton in the cavity—nothing in the roots at the first setting, cover with temporary filling (never sandarac) leaving vent hole. If no trouble after 48 hours, dress again with cotton twists in root-canals, saturated with creosote, repeat until all soreness and oder has disappeared, then fill canals with chloro-percha or gutta-percha points. If the pulp is putrescent treat with sodium and potassium converting the putrescent mass with a soapy compound. Wash out with warm water, followed by pyrozone 3 per cent. with equal quantity 1-1000 bichloride mercury. Dress as before. Counter-irritation by applying iodine, saturated solution, on the gum or a small patch of adhesive plaster warmed and powdered hydrate of chloral pressed into the surface, placed over the root left on some time. Hot foot bath at night with antikammia 5 grain caffeine 1 gr. every two or three hours. Saline cathartics are also strongly advisable. If the inflammation is persistent, force the abscess, putting a hot raisin or fig over the root. If a fistula is present sterilize the canal, forcing through permanganate of potash 10 per cent. followed by pyrozone, then carbolic acid, 1 part; oil cassia, 2 parts; oil eucalyptus, 3 parts. Repeat several times with 24 hours interval, then force through chloride of zinc (40 grs. to the ounce). If it is found difficult to force the medicament through with the syringe, fill the root canal cavity, saturate a piece of absorbent cotton with the same and place in the cavity, cover with a piece of rubber and force down with "ball puncher"(?) In case the tooth will not tolerate a close dressing, pass a probe in the root, pack stopping around and when hard withdraw the probe. This allows gasses to escape but does not permit enterance of saliva. Immediate root-filling, after removal of live pulp, Dr. Young considers objectionable, as the tooth breaks down much sooner than when the dentine has been thoroughly sterilized. When ready to adjust the rubber dam, pierce the holes with a red hot needle (an old syringe needle is good). This makes a hole less liable to tear, and hugs the teeth closer. For filling root-canals Dr. Young prefers chloro-percha and gutta-percha points—cement absorbs and becomes offensive; lead discolors; gold is too difficult. If the tooth is off color, after the root is filled, with the dam still on, fill the cavity with pyrozone 25 per cent; evaporate with chip blower (—or pipette with the bent point drawn out smaller by heating in alcohol flame till red hot and drawn out to size wanted), or asbestos may be used as a vehicle for applying the pyrozone. Open the pyrozone tubes with caution, cooling thoroughly in ice water and holding in towel wet with ice water while grinding or filing off the end. In filling proximate cavities in molars and bicuspids, a convenient matrix may be made from a strip of very thin copper, about 38 gauge. Cut a piece just the width of tooth to be filled and long enough to go half way around the tooth. Anneal and place, binding it to the tooth with ligature thread passed around several times. Then with bibulous paper, cotton or spunk, pressed in cavity outward against the matrix, give it the required contour, seeing that it fits snugly at cervical margin.

In closing Dr. Young addressed an emphatic warning against the use of coagulants in the treatment of teeth.

In the discussion, being asked why he so emphasized this, he said—There is something there that you want to get out. If you build a wall around the enemy's camp, and they have full supplies of everything they want, you will never capture them! If you seal up, cover in, encapsule the contents of the tubuli, you prevent the entrance of agents with which to sterilize the dentine. You do not want to seal in the decomposing matter. Coagulation makes an impenetrable bar to your remedies.

Dr. Conley thought that if cement became offensive in the tooth it was because the tooth had not been properly disinfected.

Dr. Young attributes it to osmosis.

Dr. Stubblefield considers gutta-percha open to the same objection. In a dead tooth, through endosmosis moisture is always present, stasis resulting in decomposition.

Dr. Young cited the Atlantic cable as proof of the impracticability of gutta-percha.

Dr. Stubblefield thought the conditions under which gutta-percha is used in a root canal, and at the bottom of the Atlantic Ocean differed too widely to admit of comparison. A dead man tied down where the Atlantic cable lies would be pickled and preserved forever. He thinks the apical foramen should be closed by something absolutely non-absorbent, and for this purpose uses amalgam, carrying it up to the apex on a suitable instrument and packing it there. Withdraw all you please and you will still leave a little button—a lock on the back door.

Dr. Stubblefield thinks Dr. Young's treatment too complex, requiring too great a variety of agents. He thinks treatment is too often overdone. He commends taking off pressure on a sore tooth, the equivalent of placing a broken arm in a sling, giving it a rest. He considers creosote to be an escharotic, and therefore a coagulant, and consequently, on Dr. Young's own theory is not the thing for use in a root canal. Dr. Young does not consider it either escharotic or coagualant, having demonstrted that.

Dr. Stubblefield: Then I have long labored under a delusion and am glad to have it dispelled. Dr. Stubblefield advocates drilling out root-canals, removing all infected dentine till sound structure is reached, using a large Morey or Gates-Glidden drill at the mouth of the canal; enlarging cautiously, and using smaller drills as the canal is penetrated, learning by experience to *see* with the touch, and having sufficient knowledge of the anatomy of the teeth to be able proximately to know when nearing the apex.

Dr. J. P. Gray is very cautious about drilling into unknown territory. He advocates the use of cement as being the best thing there is for the tooth, as long as it stays in. A good quality of cement will save a tooth longer than gold, especially if the cervical margin is lined with tin. Dr. Gray uses gutta-percha over the tooth adjacent to the sore one, but cools it in the mouth, first brushing the tooth with chloroform to make it adhere. If the walls of a cavity are smeared with the liquid that comes with cement, you will secure absolute union between the cavity walls and the cement. It is good in saucer shaped cavities and is equally good when using cement for filling crowns. He considers Justi's cement the only reliable American cement on the market, and sees no reason why fillings of this cemont should not stay in for years.

The secretary read an essay entitled "The Ideal Professional Man," contributed by Dr. B. D. Brabson, Knoxville, Tenn.

He said: * * * Man's inherent right to become superior in his vocation is limited only by his desires and capabilities for no allegation is more positively proven than "As a man thinks so is he." A man can rise no higher than his own conception of his possibilities, and for a man to arrange his plans of life without regard to his right to excel is detracting from the ideal. * * *

The truly great man is he who is able to adapt himself to his surroundings, to rise above his failures, and whose resolution grows strong in its encounter with difficulies. * * * * We must measure success or failure by the good we do.

The subject of Ethics was discussed at some length in connection with charges preferred against two members of the association, and especially the importance of having the Code of Ethics read in the presence of those about to join the association, that they may know to what they bind themselves.

By resolution the Constitution, By-Laws and Code of Ethics was ordered incorporated, as an appendix, in the published volume of transactions, so that every member of the association would be placed in possession of it in permanent form. When sent out in the form of a letter or pamphlet they are liable to be thrown aside as perhaps "another local anæsthetic circular" and consigned to the waste basket without having the wrapper removed.

The report of the Board of Dental Examiners showed that of twenty-four applicants, thirteen had been granted license. A recommendation from the board in regard to efforts to secure the passage of an amendment to the dental law prohibiting the extraction of teeth except by licensed practitioners of dentistry or medicine, elicited a lengthy discussion of this point. The matter was finally referred to a committee of three, to be put into proper shape for action at the next meeting of the association.

The report of the Publication Committee showed that the advertising pages in the volume of transactions covers the cost of publication of the proceedings in this desirable form.

ELECTION OF OFFICERS.

President, Dr. A. A. Pearson, Montgomery; First Vice-President, Dr. P. R. Tunstall, Mobile; Second Vice-President, Dr. F. H. M'Anally, Jasper; Secretary, Dr. J. H. Crossland, Montgomery; Treasurer (re-elected), Dr. G. M. Rousseau, Montgomery.

Selma was chosen as the next place of meeting, the time being always the second Tuesday in April.

